# Assessment of Hearing or Vision Impairment in Studies Collecting Cognitive Data in Older Adults

**DOI:** 10.1093/geroni/igaa057.2996

**Published:** 2020-12-16

**Authors:** Chelsea Liu, Lama Assi, Niranjani Nagarajan, Danielle Powell, Kening Jiang, Dillan Villavisanis, Bonnielin Swenor, Jennifer Deal

**Affiliations:** 1 Johns Hopkins University, Baltimore, Maryland, United States; 2 Johns Hopkins Wilmer Eye Institute, Baltimore, Maryland, United States; 3 Johns Hopkins University School of Medicine, Baltimore, Maryland, United States; 4 Johns Hopkins School of Public Health, Baltimore, Maryland, United States; 5 Icahn School of Medicine at Mount Sinai, new York, New York, United States; 6 Johns Hopkins Bloomberg School of Public Health, Baltimore, Maryland, United States

## Abstract

Despite the high prevalence of sensory impairment in older adults, there are no standard practices for its consideration in cognitive studies. We conducted a systematic review to identify and survey prospective cohort studies collecting cognitive data in older adults in order to determine whether and how hearing and vision were considered. Among 81 cohorts that responded, 30 (37%) objectively assessed hearing, with audiometry as the most frequently-used method; 61 (75%) used patient-report and 12 (15%) used provider-report to subjectively assess hearing. Forty-one (51%) cohorts objectively assessed vision, half of which measured distance or near visual acuity; 55 (68%) used patient-report and 10 (12%) used provider-report to subjectively assess vision. Nineteen (23%) cohorts offered hearing accommodations and 30 (37%) offered vision accommodations during cognitive testing. Findings indicate variation in methods used to assess hearing and vision as well as in accommodation practices that could impact estimates of cognition among older adults.

